# Judicial Department

**Published:** 1856-10-01

**Authors:** 


### Part Fourth.

### Judicial Department.

### ANNUAL MEETING OF THE ASSOCIATION OF MEDICAL OFFICERS OF ASYLUMS AND HOSPITALS FOR THE INSANE.

THE stated Annual Meeting of the above Society was held on the 1st August, in the Derby County Asylum, at Mickleover, presided over by Dr. Hitchman, the Physician Superintendent of that establishment. The attendance on the occasion was very large and influential: amongst those present were Dr. Campbell, Essex Asylum; Dr. Thurnam, Wilts Asylum (who, however, was obliged to leave before the regular business commenced); Dr. Forbes Winslow, London; Dr. Robert Stewart, Resident Physician, Belfast District Asylum; Dr. Sherlock, Resident Physician of the Worcester County Asylum; Dr. Tuke, Chiswick; Dr. Caleb Williams, York; Mr. Walsh, Surgeon Superintendent, Lincoln Asylum; Mr. Cornwall, Fairford Retreat, Gloucestershire; Dr. Dickson, Resident Physician, Manchester Royal Asylum at Cheadle; Mr. Buck, Medical Superintendent, Leicestershire Asylum; Dr. Mackintosh, Dinsdale Park, Darlington; Dr. McCreipt, Assistant Resident Physician, Somersetshire Asylum at Wells; Mr. Ley, Medical Superintendent, Oxfordshire and Berkshire Asylum; Dr. Lockhart Robertson, London; Mr. Brushfield, Medical Superintendent, Chester County Hospital for the Insane; Dr. Davey, Northwood; Dr. Prichard, Abington Abbey; Dr. Hewson, Physician Superintendent, Coton Hill Institution for the Insane, Stafford; Dr. Bucknill, Physician Superintendent of the County Devon Asylum at Exminster; Dr. Boisragon, Physician Superintendent of the County Cornwall Asylum; Mr. Isles, Cirencester; Dr. Seaton, Sudbury; Dr. Warwick, Salisbury; Dr. Paul, Camberwell House; Mr. Eddison, Nottingham; Mr. Millar, Stone; and Mr. Stilwell, jun., Mickleover.

Several letters of apology for non-attendance were read by the President, and amongst them from Dr. Conolly, of Lawn House, Hanwell; Dr. Kirkman, Suffolk; Dr. Flynn, Clonmel, Ireland; Dr. Williams, Gloucester, &c. &c. Much sympathy was felt, in consequence of the melancholy fatal accident which happened recently to Dr. Williams, the Visiting Physician of the Nottingham County Asylum, who was to have been present, but for his sudden and lamented removal.

The minutes of the last annual meeting held in London were read and confirmed.

Dr. CAMPBELL read the Auditors' Report, which stated that the accounts of the Treasurer had been audited and found correct, with a balance to the credit of the Association.

Mr. LEY entered into a lengthened statement of the past and present condition of the Association as to membership, by which it appeared, that during the past year one hundred members had paid their subscriptions, and that several more would still, it was likely, do so. The Association, he observed, was in a very solvent and flourishing state, financially and influentially, and had every prospect of being increasingly so.

Dr. L. ROBERTSON read a list of new members, thirteen in number, now proposed to be balloted for; also of the following four honorary members, viz., Sir Benjamin Brodie, Bart., Dr. Holland, Mr. Wilkes (late Medical Superintendent of the Staffordshire Hospital for the Insane, now one of Her Majesty's Commissioners in Lunacy), and Dr. Peach (one of the Visiting Magistrates of the Derby County Hospital for the Insane).

Dr. Forbes Winslow having inquired if Mr. Wilkes desired being still connected with the Association, the President read a letter from him, the purport of which appeared to remove all doubt upon that head.

A long discussion now ensued as to the shortest method of proceeding with the ballot for so many, the time of the meeting for general business being very limited. Dr. Forbes Winslow and Dr. Caleb Williams moved a resolution to the effect that *pro hac vice* the candidate members, ordinary and honorary, be elected by ballot in the mass, and not individually, with the view of saving time. This course, however, being strongly objected to, as being likely to establish a bad precedent, an amendment was moved by Dr. Davey, seconded by Dr. R. Stewart, that a ballot be taken for each proposed member separately, which being pressed to a division was lost, and the original resolution carried by a large majority, when the several candidates on the list were unanimously elected.

Dr. Tuke then gave notice that he would at the next annual meeting move that the names of candidates for honorary membership be sent in in sufficient time to the Secretary of the Association in order to appear in the printed notice issued to members for the annual meeting.

Mr. Ley now moved, in very complimentary terms, that Dr. Forbes Winslow be the President elect for next year, observing that no member of their important branch of the medical profession had done more to advance its interests, and support its high character and independence, than Dr. Winslow.

Dr. L. Robertson seconded Mr. Ley's motion, stating that this was an honour which accidentally devolved upon him, as Dr. Sutherland, had he been present to-day, intended to have done so, and in his stead Dr. Bucknill, who, however, had not yet made his appearance, which was to be regretted.

The President having put the motion from the chair, it was carried *nem. con.*

Dr. Forbes Winslow returned thanks for the enviable honour and position now conferred upon him, and assured the meeting that he most fully appreciated both, and that nothing should be left undone on his part to promote the increased usefulness and the high and important objects of their invaluable and influential Association.

It was now arranged that the annual meeting for 1857 should be held in London, the exact time and the place to be for future arrangement. It was suggested, however, as regarded place, that the Freemasons' Hall should not be selected, it being deemed a very inconvenient locality.

Mr. Ley was re-elected Treasurer; Dr. L. Robertson, General Honorary Secretary; Dr. Robert Stewart, of Belfast, and Dr. W. A. F. Browne, of Dumfries, Honorary Secretaries for Ireland and Scotland respectively; and Dr. Campbell and Mr. Prichard, Auditors; all for the current year.

Dr. Forbes Winslow, seconded by Dr. Prichard, moved that Dr. Bucknill be reappointed the Editor of the Association's Journal, which he did with great pleasure, the able manner in which it had been conducted reflecting the greatest credit upon Dr. Bucknill and the Association. The motion having been put from the chair, was carried unanimously.

A short adjournment now took place, during which the members were entertained at a splendid luncheon provided by Dr. Hitchman; which having been done ample justice to, the several wards of the establishment were visited and minutely examined, as also the farm and grounds, all of which afforded the utmost satisfaction, each department of this extensive hospital affording the fullest evidence of the greatest care and skill being exercised in the conduct and arrangements.

The meeting having been again constituted by the President resuming the chair, Dr. Winslow called attention to the extraordinary circumstance, in this age of advance and progress, in the treatment of the insane—so far as regarded the district hospitals for the insane in Ireland—that to the present time no authoritative provision was made in them for their being superintended by gentlemen of the medical profession exclusively, as was the case in England, Scotland, all over the Continent, and in the United States of America. True it was that the Lord-Lieutenants of Ireland had, for some years past, according as vacancies occurred in the public asylums in Ireland, exercised the powers vested in them by Act of Parliament, by appointing none but physicians to preside over those important institutions; but this was more from the force of public opinion and other accidental circumstances than from any legal obligation imposed on their Excellencies to do so, who might, if they pleased, appoint their valet or their butler to so responsible a charge, and be blameless in, he admitted, such an unlikely event, so entirely was a free choice left in their hands. He (Dr. Winslow) need only refer, in proof of this, to a Bill before Parliament last session, known to them all, having been published in the medical periodicals of the day, for "Amending and Explaining" the Acts for the Regulation of the District Asylums in Ireland, in which power was to be continued to be given to the Irish Executive Government to appoint "managers, matrons, and visiting physicians," by which it would be seen that a *resident* medical officer was entirely overlooked, if not purposely ignored, instead of being unmistakeably and most distinctly provided for. What the term "managers" meant was a mystery to them on this side of the Channel; but perhaps their respected member, Dr. Stewart, whom he was happy to see amongst them to-day, could enlighten the meeting on that head: if intended to mean duly-qualified members of the medical profession, it was anything but a flattering title to their body, and should give place to a more correct and less offensive designation. In fact, the time had fully arrived that this Association should express its mind upon the subject; and with this view he would, without further preface, move the adoption of a resolution to the effect that it be strongly urged that, in the Bill announced officially to be introduced into Parliament next session in regard to the Irish asylums, it be made legislatively compulsory to appoint none but members of the medical profession as the chief resident officers of those important public institutions.

Dr. Seaton said that, fully concurring in all that had been so well stated on this very important matter by Dr. Winslow, he cordially seconded the resolution.

Dr. Stewart, at the special call of the meeting, begged to state that the case was precisely as Dr. Winslow had mentioned, there being no legal obligation binding the Irish Executive to commit the superintendence of the district asylums in Ireland to medical men, but that, since the year 1843, no instance had occurred of any but physicians being placed over them as vacancies arose amongst the lay officials quaintly styled "managers." This new order of things had its commencement in the Belfast Asylum, and in his own person; for when he (Dr. Stewart) was appointed, some years previously, to that establishment, his being a member of the medical profession was an accidental circumstance, and not required, if not actually a fault; his duties being simply "moral," and not at all in a medical capacity. In the year referred to, however, viz., 1843, Lord St. Leonards, then Sir Edward Sugden and the Chancellor of Ireland, seeing that changes were requisite in the internal *régime* of the Irish asylums, induced the Privy Council to adopt an entirely new code of rules and regulations for their government, which, though an improvement in many important respects on the old ones, were found impossible to be carried properly out in their new professional aspect unless by a medical man residing in the asylums. The Governors of the Belfast Asylum in this difficulty applied to the Government to permit their resident officer to act *medically* as well as morally, and to be recognised as the "Resident Physician" of the institution, which was at once agreed to, thus getting accomplished a former earnest suggestion of the Governors to the Executive, that a medical practitioner only should have the immediate conduct of an hospital for the insane. It was thus that their profession obtained a *locus standi* within the walls of the public asylums in Ireland, and not by any legal enactment, as most assuredly ought to be the case, however good and liberal the acts of the Government might have been of late on this head.

Mr. Ley strongly supported the necessity of the resolution, and referred to a proposition of his at a former meeting of the Association, that a standing committee should be appointed to watch over all measures before Parliament relating to lunacy, and act according as circumstances required.

After some further remarks, the resolution was unanimously adopted, and copies of it directed to be forwarded to the chief secretary of the Lord-Lieutenant of Ireland and the Irish inspectors of hospitals for the insane, and also that a deputation should wait at the Irish office in London on the chief secretary, during the ensuing session of Parliament, to urge the requirement suggested in the resolution being embodied in the proposed Bill to be then brought forward for amending and explaining the Acts in connexion with the public establishments for the insane in Ireland.

Mr. Buck made some pointed observations, incidentally arising out of the state of the Irish asylums, upon the studied avoidance of anything approaching to the medical element in the last report, as well as the reports generally of the Commissioners in Lunacy, which he considered very remarkable in such important official documents.

Dr. Lockhart Robertson, after some forcible and well-received remarks, moved a resolution pressing the proper authorities to take immediate steps for the due establishment of a military hospital for the insane.

Dr. Davey, in seconding Dr. Robertson's resolution, in the spirit of which he said he heartily concurred, stated that having had much intercourse with military medical men, he in that way knew that the necessity was urgent for a military asylum.

The resolution was carried unanimously.

Dr. Caleb Williams, of York, referring to what Mr. Ley had said to-day about a standing committee to watch the progress of actual legislation in lunacy affairs, and it forcibly striking his mind that such a committee would be of the utmost importance to be in existence to unite with and move along in every possible way with the Commissioners in Lunacy, not be in antagonism with them, now proposed that such a committee be appointed, consisting of the President elect, Mr. Ley, Dr. Robertson, Dr. Sutherland, and Dr. Conolly.

Dr. Seaton seconded the resolution (whose name had been included in it, but was subsequently withdrawn on the ground simply that the committee should not be too large).

Dr. Winslow said that he deemed of the utmost importance the actual existence of a committee such as was now suggested, and mentioned facts which came within his own knowledge during the progress of former legislation in lunacy, of the serious want of such an authorised committee, and the exertions he himself had personally to use, to have details of the greatest moment corrected during the progress of bills in connexion with their specialty.

Dr. Bucknill (who joined the meeting shortly before this stage of the proceedings) observed, in supporting the resolution, that at the recent general meeting in Birmingham of the Provincial Medical and Surgical Association, from which he had just arrived, Mr. Spooner, M.P., pointedly stated that when measures in regard to medical legislation were before the House of Commons, he and the other private members were left so entirely in ignorance of the profession's wishes in those matters of detail wherein their own interests were so mainly concerned, that it was impossible, under such circumstances of apathy and indifference, serious defects in legislative proceedings of a professional character would not ensue.

The resolution, after some further remarks from Dr. Williams, Mr. Eddison

(who suggested that the committee should, if necessary, call a special meeting of the Association), and others, was adopted unanimously.

Dr. Bucknill, in laudatory terms, now moved a resolution expressing the satisfaction of the Association at Mr. Lutwidge's elevation to be a Commissioner in Lunacy.

Dr. Winslow seconded it, and stated that for several years before the Lunacy Commission existed, of which Mr. Lutwidge had been its first and most efficient secretary, he (Dr. Winslow) had been on terms of private intimacy with him, during which he always found Mr. Lutwidge a man of the highest honour and worth.—Resolution carried.

The case of the convict Dove having been submitted to the Association by Dr. Davey, who considered him not responsible for his acts, a long and desultory debate ensued, which, however, ultimated in no practical result. After the transaction of some further routine business, the chair was vacated by Dr. Hitchman, when a cordial vote of thanks was passed to him for his able and urbane conduct as President, as well as for the hospitable and sumptuous entertainment provided for the Association. The Visiting Justices of the asylum were also thanked for their permission being given with the best spirit to the Association to hold their annual meeting on the present occasion in their asylum, thus affording the opportunity of seeing all its departments, the completeness of which, in every respect, and the high order in which the institution was kept, elicited the warmest commendation of the Association.

In the evening the Association, with several invited guests, dined together, according to custom, Dr. Hitchman being in the chair. This was an exceedingly social and pleasant finale of the day's proceedings, the interchange of good feeling and friendship being abundant, and the speeches delivered on the occasion, especially by the President, being of a high order of eloquence and ability.

---

### THE CONFESSION OF DOVE.

"I left the farm at Whitewell, near Barnham, in February, 1855, and then went to reside at Normanton. I continued there until the 21st of December, 1855, at which time I removed to Cardigan-place, in Leeds. In September, 1854, I had some conversation with one John Hardcastle, who was employed as farm-labourer at Whitewell, about 'wise men' being able to find out thieves. I had shortly before that lost a dog. He said they could, and mentioned Harrison, of the South Market, in Leeds, as being a wise man. He then stated, as an instance, that he (Hardcastle) had heard that the bailiffs were going to enter his house with an execution; that he went over to consult Harrison, and to get him to do something to keep them from coming; that Harrison had caused the horse which was being driven by the bailiffs to take fright; that they were thrown out and injured so much, and were confined to bed so long, that he, Hardcastle, was able to remove the goods out of his house. Hardcastle lived about Meanwood at this time; he went home every Saturday night, and returned on Monday morning. He also stated that on a former occasion two guns had been stolen from some place at or near Roundhay, where he was then working; that he consulted Harrison, and Harrison told him that he would cause one of the men to pass by him the next evening and shoot a rabbit; that a man did so the next evening, and he was apprehended. I had at that time given a notice of my intention to quit my farm, and I was desirous of retaking it of Mr. King, the steward. I talked of this to Hardcastle, and he told me that Harrison could so work upon King as to induce him to let it me again. I then desired Hardcastle to see Harrison, and told him that the next time I went to Leeds I would see him with him (Hardcastle). We were mowing the barley at the time when this conversation took place. Hardcastle afterwards told me that he had seen Harrison, and that Harrison would be glad to see me. In about a month after this I went over to Leeds with Hardcastle. We had some potatoes to take to the market at the time. After selling the potatoes we went to see Harrison. We met with him at his own house; this was about noon. He arranged to meet us at the Old Red Lion Inn (Orrell's), which is in Meadow-lane. He did meet us there almost immediately. We were in a room together there until about six in the evening. I told Harrison, in the presence of Hardcastle, that I wished to retake my farm, and Harrison then promised to come over to my house and see about it. He did make some arrangement as to when he would come, but I forget the time; my impression is that there was no particular conversation about any other matter that afternoon. I gave him the date of my birth to work on my nativity. We sat drinking together, and I paid for a good deal of it. In about ten days after this, Harrison came over to the Fox and Grapes Inn, in Kiddal-lane, near my farm, with Mr. Orrell, of the Old Red Lion. Harrison came to my house about nine o'clock in the morning. He left Orrell at the Fox and Grapes. I met Harrison near the gate leading out of the turnpike-road. Hardcastle was with me at the time. I then said to Harrison, in the presence of Hardcastle, 'I shouldn't like Mrs. Dove to know that you are here.' Harrison said that he would make all right. We then went into the fold-yard, and thence into the granary, on the eastern side of the buildings. Hardcastle did not accompany Harrison and myself into the granary. When I gave Harrison the date of my birth, at Orrell's, I understood that Harrison required that information to work on my nativity, before he could induce King to let me the farm again. When we got into the granary he pulled out a mahogany box, with a large mariner's compass in it, to see what were the cardinal points in which my house stood: after doing this he brought out of his pocket five little pieces of copper of the shape of halfpennies; on each of these he marked a kind of hieroglyphical form. I then asked him what those were for. He said, 'Your house and farm are bewitched; I'll show you what they are for.' I then turned round and gave him a few potatoes (three) of a large kind of sets; they were called flukes. We then went downstairs, and as we were going down Harrison slipt one of the copper pieces in between the steps leading out of the gangway into the fold-yard. When we got down the stairs, Harrison asked me if there was any way into that yard except by the large fold-gates. I told him there was a road through the cowhouse. We then went through the cowhouse and through a door opening into a shed; and while in that shed he, Harrison, turned and looked round, and then threw one of the copper pieces on to the wall-plate between the cottage and the shed, and then said, 'That's all right.' We then went round the end of the wagon-shed to the gate where I first met him, through that gate across the road to a gate opposite, at which place he produced another of the copper pieces, and he there placed that copper piece between the gate-post and the soil. We then returned to the first gate, where he produced another, which he placed between the gate-post nearest to the orchard and the soil there. When he had done that he commenced to pray, at the same time leaning his head upon his arm, which was then upon the gate-post. He prayed aloud, so that I could hear him. I do not remember the words, but it was a prayer in which he referred to the seven wise men, and of which he was one, and it was to free me and the farm from witchcraft. His prayer occupied about five minutes. When he had concluded, he said, 'There, you are all right now; no one can pass this gate to do you harm.' I then asked him where was the other coin. He said it was in his pocket, but that it must be planted in the house. We then went into the house, and I then introduced him to my wife as a dental surgeon, and as having known him some time. I then asked him to stop to dinner, and he stayed. Before dinner, I and Mrs. Dove showed him round the house, and as we came down stairs Harrison told me that he had planted the other copper piece. My wife was then in the bed-room. I drew some beer, and whilst we were sitting in the common sitting-room my wife passed through that room into the kitchens, and Harrison then said to me there, 'No person will ever molest you,' and at the same time asked for pen and paper. I got them for him, and he wrote some hieroglyphical signs, and gave them to me, saying, 'If you want to retake the farm, and you put that paper in your pocket, you may then go to Mr. King, and he will let you it; only, you must let me know beforehand when you are going.' I put that paper in my pocket. Mrs. Dove then came into the room, and the conversation then ceased. In a short time Harrison made some remark about my wife's health,—she then looked sickly and weak. I told him that she had no appetite, and that she was never out of the doctor's hands. He then said to her that if she would only use herbs she would soon get well. He mentioned the herbs, and I was to obtain them at the druggist's, which I afterwards did. After dinner I accompanied Harrison, at his request, to the Fox. Hardcastle was also then with us, and as we were going we met Orrell. As we were going I inquired of Harrison if he had written out my nativity? He said that he had not done it, but that he would. He had something to drink at the Fox. We stayed there until about three o'clock in the afternoon. I sat next to Harrison, and I gave him then half-a-crown. The other parties could not see me give him the half-a-crown. I next saw Harrison in two or three weeks after he had been over at the farm: this was at his own house. I then told him that I had not been able to see Mr. King, and that I had lost my paper, and I asked him to give me another. He gave me another paper, and it was then agreed between us that I should see Mr. King on the following Wednesday. I then told him that I and Mrs. Dove were unhappy together; but at this time some other person came into Harrison's, and he said, 'We'll have a glass together at Orrell's, and talk the matter over.' I went there, and he promised to follow immediately. He came there shortly afterwards, I and Harrison, and remained there about half an hour. At this interview I repeated the remark that I and Mrs. Dove were very unhappy together, and he replied, 'No wonder. Mrs. Dove is always vilifying and backbiting you to her friends, and she is two-faced.' I then asked him if anything could be done so that we might live happily together, for I had married her for love and nothing else. He immediately said, 'That can be done; but it will take some time to work it round.' I asked him to do it, and he promised, saying, I might go home and be content, for he would cause her to meet me with a smile. He then asked me what I was going to give him for coming over to my house, and I told him that if he would let Hardcastle know what it had cost him I would pay him (Harrison) the next time I saw him. I then left him and went to the Black Swan Inn, at the bottom of Lowerhead-row, in Leeds (Mr. Swales's), for my horse, which I had left there in the morning. I got my horse, and at this time the ostler's wife at that inn told me that some sacks had been stolen from the inn-yard, and which were under her husband's charge. I then said to her, 'I know a man, or a wise man, who can tell you who has stolen them.' This was during the time that her husband was getting my horse out of the stable. When he came I took him into the house (that is, the house occupied by the ostler), and I took down the name of the owner, and number of the stolen sacks. I then mounted my horse with the intention of going to see Harrison, for the purpose of obtaining the information as to the thief. I started, and as I was passing Orrell's my horse stumbled and fell. I was thrown over the horse's head; my right arm was dislocated. I then went into Orrell's and got a bed there. Harrison came there, I believe, at Orrell's request. Harrison proposed to set my arm, but I preferred having our own surgeon. Mr. Morley's assistant came and set it. I had no further conversation with Harrison that evening. He was offended because I would not let him set my arm. I was removed home the same evening in a cab. In a short time after this, Hardcastle informed me that Harrison expected half-a-sovereign. Hardcastle was going to Leeds with potatoes for sale, and I ordered him to pay Harrison that sum out of the money which he would receive. I was afterwards informed by Hardcastle that he had paid it. In about ten days after my accident, I sent Hardcastle with a sample of wheat to sell in Leeds market, and which he sold to Sutcliffe and Hartley, of Bradford; but they never paid for it. The wheat was delivered on the Thursday, and the price was to have been paid on the Tuesday following. I went over on that Tuesday, but Sutcliffe and Hartley were not there; they had left word at the New Inn, Vicar-lane, that they would meet me on the following Tuesday. On that Tuesday I went with Hardcastle, but as they did not meet me at the New Inn, we went to Harrison's to request him to try to get the money. He went with us to several public-houses, but could not find the parties. He then said he would influence the parties to meet us on the following Tuesday, and pay the money. He said he would lay a spell upon them, which would cause them to meet and pay the money. I can't state whether Hardcastle heard this or not. On the following Thursday, Hardcastle was in Leeds, and saw Harrison. Hardcastle stated, on his return home, that Harrison and the parties would meet at the Talbot Inn on the following Tuesday. On that day Mrs. Dove accompanied me to Leeds in the phaeton, and Hardcastle went with a cartload of potatoes on the same day. We stayed at the Black Swan Inn. I and Mrs. Dove went to the Talbot at two o'clock. Harrison and Hardcastle came in there at that time; we waited some time, but the parties did not come. We then went to several public-houses to search for them. Mrs. Dove complained of fatigue, and said she would follow us.

"Harrison and Hardcastle went with me into the Ship Inn, and we there met with Hartley, who walked away. Harrison promised to get the money, but never did.

"About the back-end of October, 1854, my poor father was very poorly indeed. I then went to Harrison, and told him so. I asked him if he would get better; he said, 'No.' I then said to him, 'Do you know when he'll die?' Harrison answered, 'Between November and February.'

"In about a month afterwards I called upon Harrison again, and spoke about the disagreement with my wife. He then told me she never would be right until she had children, and that I had missed my mark by marrying her at all. He also said, if she would continue the medicine which he had mentioned—I mean the herbs—it would soon cause her to breed, and she would then be all right. He inquired if she had taken any of it. I told him that she had taken some little, but she would not take any more. He then said, Go home, and he would make all right.' After drinking more together, I left him.

"I met him at Orrell's accidentally in December, 1854. I told him that my father was not getting any better—could he tell me particularly when he would die? He said he could; and I then asked him if he would, and he then answered, 'Yes; he will not live till the 25th of December.' Nothing further passed at that time that I remember. My father died on the 24th of December in that year; but after the interview which I have just mentioned, and before my father's death, I had seen Mr. King, who would not let me have the farm. I, in consequence of this, saw Harrison. This was about ten days before my father's death. I told him of Mr. King's refusal, and that at the time he refused I had the paper in my pocket. Harrison said, 'Never mind; work your land as usual; he will let you the farm yet; he has a spell upon him; he is an Irishman, and will take a good deal of working upon; but rather than you should miss the farm, it will be death to him.' At this interview I rather doubted Harrison's power; but my father's death happening before the 25th of December, I was impressed with a strong belief that Harrison was possessed of supernatural power.

"I left the farm in March, 1855, and went to reside at Normanton.

"About August, 1855, I had some unpleasantness with my wife, and I went over to Harrison at Leeds. I told him of it, and he promised to make it all right. He told me that I must let him know by letter how things went on. In two days after this I wrote to him, stating that my wife was no better, and that he must do something to cause peace. I sent this letter by a neighbour named Fisher, who was a porter at the station, to post. Mrs. Dove knew that I had written a letter, but didn't know what about. She therefore sent the servant to Fisher's to get it back. She did so. Mrs. Dove then took my letter out of the envelope, and sent a blank sheet of paper instead. I was not aware of this at the time; but hearing some whispering between Mrs. Fisher, Mrs. Dove, Miss Susan Jenkins (who was then staying with us), and the servant, I suspected there was some deception about the matter. I then wrote another letter to the same effect to Harrison, and posted it myself the same day before two o'clock. At three o'clock I went by train to Leeds to see Harrison. When I arrived, he had received the envelope with the blank sheet of paper therein and my letter. He inquired the reason of the blank sheet of paper, and I told him. I then again informed him of my unpleasantness and unhappiness with my wife. He then said, 'You never will have any happiness until she's out of the way.' I said, 'How do you know that?' He said, 'If you'll come upstairs, I'll tell you; for I've got your nativity marked out.' I then went upstairs with him. He there took out of a drawer a sheet of paper about the size of that now used by you, and on which there was a circle drawn, with the signs of the zodiac. There were also hieroglyphical forms, opposite various figures denoting years, beginning at 27 (my age), and continuing down to 50 or 52. The figures after 27 were 32 repeated twice or three times. He referred to the forms opposite the various figures, and read from a book my destiny. He said that between the age of 27 and 32 everything would go against me—I should have nothing but misfortunes—that at 32 the sun and moon would come in conjunction (I think conjunction was the word he used), and that then everything would be in my favour; that at 32 years of age I should lose my wife; that at 32 I should marry again; that at 32 I should have a child, and that at 32 I should have an addition to my fortune, and that for my sake he did not care how soon it was here, for until then I should never be a happy man; that after I was 32 everything would go on well for a few years. He made other remarks as to different periods of my life to this effect—that at one period I should have another addition to my fortune; at another that I must be careful what I was about to avoid a lawsuit; at another, I must neither travel by land nor by water, for if I did an accident would occur; but he ultimately said I should die respected by everybody around me.

"At this interview I asked him what description of woman I should marry for my second wife? He referred again to the same paper and to his book, and then said, 'The person that you ought to marry will have auburn hair, light complexion, and a good fortune;' and he added, 'If you had married a person of this description at first, you would have done well.' He then closed the book, and we then left and went to the New Cross Inn. We had a glass or two of ale each there, and then parted.

"The next time I saw Harrison was when I had walked over from Normanton to Leeds. I had a walking-stick with me, which had been given me by William Windsor, who is now with Mr. David Holmes, the auctioneer. I cannot mention the date, but I think it was about September last. Miss Jenkins was staying with us at the time. When I got to Harrison's he saw the stick, which had my initials on it. He fell in love with the stick, and I gave it to him. My interview was in reference to my wife. I said to him—we were talking of a separation—'Would she go?' He replied, 'She knew better; but if she would, let her go.' I told him that I did not wish for a separation, but I wished happiness. He then said, 'If she goes, she will return.' We went to the New Cross Inn, and had some ale there, and then parted. My wife inquired about my walking-stick, and I told her that I had left it at Leeds. She afterwards, as I believe, informed my brother-in-law, Mr. Marsden, that I had dealings with Harrison, and that Harrison had got my stick, for in a short time afterwards I heard that Marsden had been at Harrison's with a policeman, to get the stick back from him. I heard this from Marsden, who wished me to give him an authority to Harrison to deliver up the stick. I did not give the authority, but wrote to Harrison to keep it, as I had given it to him. My wife commenced upbraiding me about giving the stick, and told me that if I would not send for the stick she would send Miss Jenkins for it, or go herself. They both went, but Harrison would not deliver it up. Shortly before they went over, and also after they returned, we heard strange noises in our house at night, after we had gone to bed. The noise was like that caused by breaking of pots, and sometimes as if some heavy package was rolled across the floor. One night I thought some person was kicking the door, but when I looked at the door there were no marks. All the noises I attributed to Harrison. I afterwards told him so, but he denied it. On the next day, after Mrs. Dove and Miss Jenkins had been for the stick, I gave Miss Jenkins a letter to Harrison, requesting him to give her the stick, which he did. When the stick was returned the initials had been removed, and the letters 'H. H.' put in their place. The same evening that the stick was returned there were great noises in the house. I saw Harrison again in November about my wife's temper. He said, 'Never mind, all will soon be right; she'll die before March or before the end of February.' I am not certain which he said; but when he told me that my wife would die so soon, I said to him that he had before told me that she would die 'at thirty-two,' and he then answered before thirty-two,' but 'I did not say how much before.' We then went to the New Cross Inn, and had some ale there. Nothing further occurred at that time that I remember. We removed to No. 3, Cardigan-place, in Leeds, on the 21st of December, 1855. In a few days afterwards I went to the New Cross Inn, and Harrison came in: the newspaper was produced, and Harrison read about Palmer's case. I then asked him whether strychnia could be detected. He said, 'No, nor yet any other vegetable poison.' I then said, 'What other vegetable poisons are there that cannot be detected?' He replied, 'Digitalis, belladonna, particularly if it was crystallized.' These were all he remembered at the time. I then asked him if he could get or make me some strychnia? We had then got to our new house, and were much annoyed with cats. The house had been previously unoccupied. I wished to destroy the cats by poison. I had never heard of strychnia until Palmer's case arose. Harrison refused to get me the strychnia, and I told him that I could get it elsewhere.

"I went to him again in January last about my wife. On this occasion I gave him a book, 'The Magazine of Art.' I told him about my wife's temper, and he again said, 'She wont live long.' I told him that she was poorly then. He said, 'She'll never get better, as I told you before; she will die in February.' I had no further communication with Harrison until Thursday, the 6th of March, 1856. My wife died on the 1st of that month. On Thursday, the 6th of March, I went to the New Cross Inn, and after being there a few minutes I sent for Harrison. He came. I announced to him my wife's death, at the same time informing him that an inquest was held as to my wife's death. He inquired the reason why the inquest was held? I said, 'My wife died very suddenly, and Mr. Morley, the surgeon, cannot account for it, and it is known that I had strychnia in the house. Mr. Morley thinks some might have been spilt, and my wife have got some accidentally.' I then said to him, 'You told me that strychnia could not be detected, but I have since seen in the 'Materia Medica' that it can. What is your opinion now? Can a grain or a grain and a half be detected? For there is great difference of opinion on the subject. Professor Taylor says that it cannot be detected twenty-four hours after death in the human body.' Harrison said, 'What! have you poisoned your wife?' I replied, 'No; I should be very sorry.' Nothing further passed at this time. He left me at the New Cross Inn between two and three o'clock in the afternoon.

"On Friday, the 7th of March, while the inquest was going on at Fleischmann's Hotel, I went to the back door of Harrison's house. This was about three o'clock in the afternoon. I then said to him that the inquest was going on, that several witnesses had been called, and that I was suspected of poisoning my wife; and I then said to him, 'How will the case go? Shall I be imprisoned?' He replied, 'It will be a very difficult case; but I can work you out.' I then said to him, 'You only say you can. Now tell me, will you?' Harrison replied, 'Set yourself altogether at rest; I will.'

"I did not see Harrison again until he was examined as a witness at the Court-house, on the 12th of March, and I had no communication with him between the 7th and 12th of March. I had no conversation with Harrison on the day of his examination. I merely heard the statement which he made as a witness on that day.

"On the 26th of March last, at which time I was in custody at York Castle, I wrote a letter to Harrison for the purpose of ascertaining what would be the result of my trial. I sent this letter by a militiaman who was leaving the prison. He promised to conceal the letter in his clothing, and to put it into the post-office. I sealed the letter. I afterwards learned that this letter had been found by the officers of the gaol, who had searched the militiaman on his leaving prison. I have not sent any other letter to Harrison or any other person secretly since I was committed to prison. "William Dove.

"Taken by me—J. M. Barret.

"(Signed at 2.50—23rd June, 1856.)"

Up to Thursday, Aug. 7, Dove maintained his innocence, but on Mr. Barret calling upon him that morning he desired him to call again, alone, in the afternoon, as he wished to say something to him about the murder. Mr. Barret accordingly did so, and the unhappy man then made the following confession, and a fearful thunderstorm which passed over the Castle at the time gave to the scene a character and effect which can never be forgotten by those present:—

"The Statement of William Dove, made to Mr. Barret, his Attorney, on Thursday, the 7th day of August, 1856.

"I wish to repeat that the statement which I have previously made to you respecting Harrison is strictly true. Harrison has, during the time that I was at the farm at Bramham, and also when I lived at Normanton, and afterwards at Leeds, frequently told me that I should never be happy until my wife was dead. This was when I was pressing Harrison to put a spell upon her, so that I might live happily with her. About the end of last year, or the beginning of this, I was in Harrison's warehouse, opposite his house, and he then told me that belladonna could not be found in the human body after death, particularly if it was in a crystallized state; and he then offered to make me some, but I did not request him to do so. At this interview he stated very positively that I should never be happy until she was out of the way. I had no desire at this time to get rid of my wife. My belief was that Harrison was possessed of some supernatural power, and that he could, through some influence, compel her to live happily with me.

He kept continually telling me that I should not be happy until she was out of the way. I asked him in the month of February if he could do anything to get her out of the way, and he said he would lay her on a sick bed and she would never get better. The first strychnia was got, as mentioned by me in my former statement, on the 10th day of February last, and for the purpose of killing cats. It was not got for any other purpose, and at that time I never thought of poisoning my wife. The whole of the strychnia obtained on the 10th day of February was used for killing cats. The second quantity of strychnia was got by me, I believe, on the Thursday or Friday following, but I wont be sure as to the day. The first and second quantities of strychnia were kept in the razor-case, which was placed on the mantelpiece in my bed-room. I did not when I got the second quantity of strychnia think of poisoning my wife. I should not have got the second quantity of strychnia but for the wish expressed by Mr. Morley's assistant to have the skin of a cat for a tobacco-pouch. I had before this given a book to Harrison, and on the day that Elizabeth Fisher left our house I had considerable unpleasantness with my wife about Harrison having that book. She had a bad opinion of Harrison, and I think feared him. I had told her when at Normanton that he had predicted her death at the end of February. On the Saturday after Elizabeth Fisher left, I took the paper containing the strychnia out of the razor-case, and put it in my waistcoat-pocket. I then went to my mother's house. In the afternoon I had previously called at Mr. Morley's for my wife's medicine. It was an effervescing draught in two bottles. At my mother's that evening I took the cork out of one of the bottles and touched the wet end of it with the strychnia. I then put the cork in the bottle again and shook up the draught. Before this I ought to have stated that I had during that Saturday afternoon put a very small quantity of the strychnia, perhaps half a quarter of a grain, in some jelly which my sister Jane brought from my mother's. My wife took a spoonful, and made a remark about how bitter it was, and she then requested Mrs. Fisher to take some. She did, and then remarked it was bitter as aloes. I then took a spoonful, but did not taste the bitterness. I then stated that I did not find the bitterness, and requested Mrs. Fisher to taste again, but she refused. I then took a second spoonful, and tasted the bitterness. I did not swallow any. The remainder was thrown away at that time. It was after this that I went to Mr. Morley's and my mother's. On that Saturday evening my wife took some of the draught in Mrs. Whitham's presence. Mrs. Whitham tasted it, and stated that it was bitter. The draught was not shaken that night before it was taken. My wife did not suffer from the effects of it at all. On the way from my mother's towards home on that Saturday I threw the remainder of the strychnia away. I cannot tell you the feelings of my mind when I put the strychnia into the jelly and into the mixture. I cannot describe them. I did not think at the moment when I put it in as to its effects or consequences. On the Sunday evening following, which was the 24th day of February, I went into Mr. Morley's surgery, and, there being no person in at the time, I took perhaps ten grains of strychnia and folded it in paper. When I got home I placed it in the stable. On the Monday morning I gave my wife her draught (the effervescing mixture) about half-past nine, and at ten o'clock she had the attack as mentioned by Mrs. Fisher and Mrs. Whitham. At the time my wife took that draught she complained very much of the bitterness, and added, that she would tell Mr. Morley about it. There were three or four doses left in the bottle after that draught was taken, and I broke the bottle in my wife's presence, fearing that Mr. Morley might taste it. The mixture was changed on the Monday. The mixture then given was very bitter. On the Tuesday night, or Wednesday morning, I applied the wet end of the cork of the medicine bottle to the strychnia, as before. I think there might be from half a grain to a grain of strychnia on the cork when I put it in the bottle. I shook the mixture up. There were then only two or three doses in the bottle. I don't remember my wife having any attack on the Wednesday. She took her mixture on that day. On the Thursday I got another bottle of medicine from Mr. Morley's, and I again applied the wet end of the cork to the strychnia as before. About the same quantity of strychnia adhered as on the former occasion. The last dose of that mixture was taken on Friday night, at ten o'clock, and my wife was taken seriously ill in half an hour, but she had no arching of the back on that occasion that I recollect. Mrs. Fisher is mistaken, I think, on that point, but her statement in other respects is true, I believe. On that Friday night I got another bottle of medicine from Mr. Morley's. This mixture was directed to be taken four times a-day. I did not put any strychnia into that mixture or medicine, and I did not put any upon the cork of the bottle. Mrs. Whitham gave a dose out of that bottle in the afternoon of Saturday. The strychnia was in the stable, where I had first placed it, and there was no strychnia in the razor case on that day, nor during any part of that week. I was drinking at Sutcliffe's public-house on that Saturday, and I was more or less affected by liquor all the afternoon and evening. About three o'clock in the afternoon I went into the stable and took about a grain and a-half of strychnia out of the paper, and put it in another paper, which I placed in my waistcoat pocket. I put that strychnia into the wineglass, which contained a little water—I believe the water which was left in the glass by Mrs. Whitham after giving my wife the third dose in the afternoon; but I have no recollection as to the time when I put the strychnia into the glass. I gave the mixture in the evening, in the presence of Mrs. Whitham and Mrs. Wood, as stated by them in their evidence. I poured the mixture into that wineglass which contained the water and strychnia. I did not put the strychnia into the wineglass in the presence of Mrs. Whitham and Mrs. Wood, or either of them. I know that I put the strychnia in before, but I cannot remember how long before giving the medicine. I did not, when I gave the medicine on the occasions mentioned, think of the consequences of giving it; but when I saw my wife suffering from the attack on the Saturday night, it flashed across my mind that I had given her the strychnia, and that she would die from its effects.

"I was muddled before this, and didn't know what I was doing. When the thoughts of her death crossed my mind, I immediately regretted what I had done, and I believe if Mr. Morley had come in at that moment I should have told him what I had given her, so that he might have used means to restore her. I cannot disguise the anguish I felt when I returned from Mr. Morley's and found my wife dead.

"Palmer's case first called my attention to strychnia, but I never should have thought of using that or any other poison for the purpose of taking my poor wife's life but for Harrison, who was continually telling me that I should never have any happiness until my wife was out of the way. I felt my situation immediately after seeing my poor wife's corpse, and I then feared a *post-mortem* examination taking place within twenty-four hours after death, as I understood that Professor Taylor had stated that strychnia could not be detected after that number of hours had expired. Harrison had told me that I should marry a lady for my second wife having auburn hair and light complexion, as I before stated to you. I did think that when my wife should die that I would make Mrs. Whitham an offer of marriage at a suitable time. I did not think that what I was doing would cause my wife's death, and I did not give the strychnia with a view to destroying my wife to get Mrs. Whitham. Mrs. Whitham always treated me as a neighbour, and no more, and I wish to add that I believe her evidence against me is strictly true.

"I have not the slightest recollection of my wife ever stating that she wished her friends to have a *post-mortem* examination, as stated by Elizabeth Fisher, and I do not believe that my wife ever made any such statement to Elizabeth Fisher, or any other person. I once told my wife when she was ill, some weeks before her death, that if she died there should be a *post-mortem* examination. I said this because she told me her friends would blame me if anything happened to her. She immediately objected, and requested that I would never consent to such an examination, and added, 'My uncle Bishop will see that you have not one.'

"I continued to believe in Harrison's power for some weeks after I was committed to prison. I believed that he had the power to save me until June or July. On the day when I wrote the letter commencing 'Dear Devil,' I was in a low, desponding, and queer state. I can't describe my feelings. I during that day thought of committing suicide. The instrument which was found upon me on the search made that day would have been probably used for that purpose. In the evening of that day I wrote that letter, but I cannot tell you my feelings at that time. I did feel certain that the devil would come to me that night according to my request. I wrote that letter, but never intended it to be seen by any person. When they commenced to search me for the instrument I tried to conceal the letter, but the deputy-governor accidentally saw it, and took it away. I did not write that letter in consequence of any suggestion from any person or persons, and never mentioned that letter, or the search which had been made on the 24th of April, to Mr. Barret, my attorney, until within two or three days of my trial, and then only because he stated that he had received information on the subject on the Saturday before my trial. I have only to add that the verdict of the jury was just and correct, and that I freely forgive every person who has been concerned against me, as I hope to be forgiven. "WILLIAM DOVE.

"Condemned Cell, York Castle, August 7.

"Signed in my presence, J. M. BARRET, 5.30 p.m."

The concluding portion of the letter addressed by Dove to Mr. Barret on Friday evening was as follows:—

"I would wish to remark, that I committed the crime through the instigation of that bad man, Henry Harrison, of the South-market, Leeds. Had it not been for him I never should have been in these circumstances.

"I remain, respected sir, yours respectfully,

"J. M. Barret, Esq." "WILLIAM DOVE.

---

### STATISTICS OF CRIME.

A BLUE BOOK lately published contains tables of criminal offenders in the year 1854, preceded by explanations and calculations from the pen of Mr. Redgrave of the Home Office. It appears that the total number of commitments during the twenty-one years from 1834 to 1854 amounted to 196,864, of which 29,359 took place in 1854. The commitments of 1854 exhibited an increase of no less than 8·5 per cent., and exceed the average of the ten preceding years by 7·5 per cent.

This increase is unhappily general, only six of the English counties being exempted from its influence—viz., Yorkshire, Cambridge, Cumberland, Lincoln, Oxford, and Salop, where a slight decrease appears. In the metropolitan counties the increase was considerable—viz., in Middlesex, 13·4 per cent.; in Surrey, 12·8 per cent.; and in Kent, 11·9 per cent. Lancashire shows an increase of 8·0 per cent.; Derby, one of 24·9; Notts, one of 31·1; Leicester, one of 36·5; Warwick, one of 14·7; and Worcester, one of 13·9. In Essex and Herts—agricultural counties—the increase was respectively 13·4 and 23·2.

In North Wales a decrease of 2·0 per cent. is reported, and in South Wales an increase of 14·1 per cent. It is satisfactory to add that the increase is confined to the least grave classes of crime, and that violent offences have, on the whole, declined. In murder, manslaughter, and infanticide the aggregate decrease is 13·6 per cent. There is an increase in the unnatural crimes, but a decrease of 7·6 per cent. in rapes and attempts to violate the person. In the newly-defined offence, "assault and inflicting bodily harm" (14 & 15 Vict., c. 19), the numbers are 23·7 per cent. less. There has been an increase in burglary and violent robberies, and the robberies without violence include above four-fifths of the commitments for indictable offences; and in this large class the increase of 11·0 per cent., observes Mr. Redgrave, shows how largely the increased commitments of 1854 are made up of the less grave offences. Picking pockets forms an exception to this increase; while there is a large and progressive increase in larcenies by servants and frauds. Malicious offences against property do not comprise 1 per cent. of the total of the commitments. In forgeries, the number of commitments has been stationary, except as regards bank notes, in which offence an increase of 33 per cent. is reported, progressive for the last ten years. Coining is also on the increase, probably owing to the frequent refusal of the Mint to prosecute. There is a marked decrease of offences against the Game Laws. Perjury has been on the increase since the alteration of the law of evidence. Of the 29,359 offenders committed, 6,274 were acquitted and set free, 38 detained as lunatics, and 23,047 convicted of the crimes charged against them. Of these 23,047 convicts, 49 were doomed to death, 310 to transportation, 2,108 to penal servitude, 20,388 to imprisonment, and 192 to be fined or whipped. The decreased proportion of acquittals in 1854 is very remarkable, the increased commitments having added exclusively to the convictions. The effect of any diminution in the severity of punishment has not been confined to the offences to which it immediately referred; but has practically operated as a reduction of the whole scale of punishment. Of the 49 persons sentenced to death, only five expiated their crimes on the scaffold. It is sad to observe that the constantly increasing proportion of female committals only slightly decreased, while the actual number committed increased. In 1854, the proportion was 29·2 females to 100 males; in 1853, 29·5; and in 1852, 25·7. In 1842 it was only 21·6. In 1854, 14,673 prisoners were tried at County Quarter Sessions, 3,119 at the Middlesex Sessions, 5,394 at borough sessions, 4,571 at the assizes, and 1,512 at the Central Criminal Court.

